# Knowledge of fermentation and health benefits among general population in North-eastern Slovenia

**DOI:** 10.1186/s12889-022-14094-9

**Published:** 2022-09-07

**Authors:** Maja Šikić-Pogačar, Dušanka Mičetić Turk, Sabina Fijan

**Affiliations:** 1 Faculty of Medicine, Department of Pediatrics, University of Maribor, Taborska ulica 8, 2000 Maribor, Slovenia; 2 Faculty of Health Sciences, Institute for Health and Nutrition, University of Maribor, Žitna ulica 15, 2000 Maribor, Slovenia

**Keywords:** Fermentation, Fermented foods, Health benefits, Knowledge, Microorganisms

## Abstract

**Background:**

Fermented foods are staples of the human diet and fermentation process has been used by humans for thousands of years. The preparation of fermented foods was performed in the past without knowledge of the role of microorganisms involved. Nowadays, fermented foods, due to their proclaimed health benefits for consumers, are becoming increasingly popular. Our study was constructed to provide data on awareness and use of fermented foods among people in North-eastern Slovenia.

**Methods:**

The cross-sectional study included 349 individuals (16–89 years of age). An online survey was designed to assess the participants' knowledge of fermentation, fermented foods, the consumption of fermented foods and awareness of the health benefits. Data were collected from March to June 2021 and analyzed using IBM SPSS 27.0.

**Results:**

Compared with the youngest participants (< 21 years) knowledge of fermentation was higher in older individuals (*p* < 0.001). More than a half of the participants recognized the role of lactic acid bacteria and yeasts in fermentation process, however, only 18.3% of participants were aware of the role of the molds. Only 25.9% of the participants have become acquainted with fermented foods at home and 62.2% of them were aware of health benefits of fermented foods, but mostly on gastrointestinal health and the immune system.

**Conclusions:**

As people today live predominantly in urban areas and incline towards westernized foods, they often lack the knowledge of fermentation and awareness regarding the nutritional value of fermented foods and their preparation. Steps should be taken to educate younger generations regarding the health benefits of fermented foods especially considering that most of them expressed their interest in learning more about the process.

## Introduction

Food preservation has been important since the early days of humanity and the process of fermentation (in addition to salting) represents one of the oldest methods of preserving and producing foods and beverages [1]. Fermented foods are believed to have been a part of the human diet for almost 10,000 years and today account for nearly one-third of global food consumption [2, 3]. The term “fermentation” comes from the Latin word “fermentum” and refers to a process that utilizes the growth and metabolic activities of microorganisms that are present in plant or animal material [4]. It involves the chemical transformation or breakdown of complex organic substances or other food components into simpler compounds by the action of naturally occurring enzymes and fermenting microorganisms. The microorganisms involved in fermentation include bacteria (e.g. Lactobacillus spp., Streptococcus spp., Enterococcus spp., Bacillus spp., Acetobacter spp.), yeasts (e.g. Saccharomyces spp., Pichia spp., Candida spp.), or molds (e.g. Aspergillus oryzae, Penicillium roqueforti, Rhizopus spp.) [5, 6]. The definition of fermentation has been updated by the International Scientific Association of Probiotics and Prebiotics as “foods made through desired microbial growth and enzymatic conversions of food components’’ [6]. Most traditional fermentations are spontaneous and occur when indigenous microorganisms are allowed to grow and metabolize raw ingredients under favorable conditions. Induced fermentations, on the other hand, require addition of starter cultures (representing individual or mixture of defined microorganisms) to induce a desired change in the food substrate, assuring quality and safety [5, 7].

During food fermentation, fermenting microorganisms synthesize a vast variety of metabolites that prevent the growth of spoilage and pathogenic microorganisms, consequently extending the shelf life of the fermented product [4]. For example, LAB synthesize metabolites including lactic and acetic acid, carbon dioxide, ethanol, hydrogen peroxide, bacteriocins, and antimicrobial peptides that suppress the growth of spoilage and pathogenic microorganisms [8]. In addition to the food preserving role of fermentation, the metabolic activity of microorganisms can alter the nutritional characteristics of foods and provide beneficial effects on human health [9]. This is an important aspect of fermented foods, considering that today’s modern diet is characterized by a high intake of saturated fats, excessive salt consumption, too much refined sugar, and an overall low nutritional value. Modern lifestyles and diets with less exposure to microorganisms are both associated with changes in the human colonic microbiota that can lead to many inflammatory diseases and are the major cause of morbidity and mortality worldwide [10]. This is reflected in the increasing research and market for nutraceuticals to combat these lifestyle disorders [11]. However, a potential solution could be the simple inclusion of fermented foods in the diet. Fermented foods provide health benefits to consumers in many ways due to the presence of vitamins, minerals, and bioactive compounds with prebiotic, antiproliferative, antimicrobial, or antioxidant properties produced during the fermentation process [6, 12, 13]. The latter include γ-aminobutyric acid (GABA), which acts as a protective agent against cancer and cardiovascular disease and regulates blood pressure [14], or short-chain fatty acids (i.e., acetate, propionate, and butyrate), which may exhibit hepatoprotective and hypocholesterolemic effects [15].

In this sense, the presence of such microbial-derived bioactive compounds might help in prevention of cardiovascular disease, gastrointestinal, immune and neurological disorders [6, 16]. In addition, fermentation helps in significant reduction of antinutritional factors such as phytic acid, trypsin inhibitors, etc. [3].

Lastly, fermented foods might help restoring the imbalances between beneficial and pathogenic bacteria in the colon and serve as a vehicle for beneficial microorganisms that might have an important role in human health [10]. Certain strains of these bacteria and yeasts are referred to as “probiotic”. However, the term “probiotic” may only be used when there is evidence of health benefits conferred by well-defined and characterized live microorganisms [6]. Many uses of probiotics from fermented foods have been proposed, including modulation of intestinal mucosal immunity, prevention and treatment of intestinal infections [16], lowering serum cholesterol, suppressing the growth of pathogenic microorganisms, modulating the immune system, etc. [17]. Due to their health benefits, it has been suggested that fermented foods should be included in national dietary recommendations [18, 19]. It should be noted, however, that scientific evidence of strain-specific benefits from controlled intervention studies is required for a product to be labeled as “probiotic fermented food” with presumed health benefits. In addition, demonstrated safety and sufficient numbers of the particular probiotic strain in the final product are required to provide the claimed benefit [6]. Similarly, fermented foods with unidentified content of microorganisms cannot be considered “probiotic fermented foods” [10].

Worldwide, different products are the result of fermentation reflecting the biocultural diversity of communities across the globe. Different microorganisms (bacterial and yeast species) are involved in the process and contribute to the unique flavors, textures, and nutritional profiles of fermented foods [6, 10]. Therefore, there is a wide variety of raw materials and derived products worldwide. Traditional fermented foods in Slovenia include sauerkraut, sour turnip, fermented beets, salami, vinegar, wine, cheese, yogurt, smetana, etc. [20–22].

In Slovenia, as in other countries, traditional knowledge about fermentation was passed from older to younger generations in the past, most frequently with no understanding of the role of the microorganism(s) involved in the process [11, 20, 23]. The emergence of microbiology as a science in the mid-nineteenth century led to increased knowledge about the biological basis of food fermentations, and since then the process has been understood for the first time [24]. As a result, much is known about the process today, but due to lack of interest, traditional knowledge of fermentation is no longer passed on to younger generations as it was in the past [25]. Insufficient knowledge about fermentation, the health benefits of fermented foods, lack of interest, and lack of time associated with modern diets/lifestyles have led to a gradual decline in the use of traditional foods [25].

The present study aims to assess knowledge about the preparation of fermented foods and awareness of the health benefits of fermented foods among people of all ages in northeastern Slovenia.

## Methods and data

The present study utilized a cross-sectional observational design employing a survey method. The questionnaire was designed very broadly in order to shed light on all the important issues related to people’s attitudes and knowledge about fermentation. Our main objectives were to analyze 1) participants' knowledge about fermentation and fermented foods, 2) consumption of fermented foods, and 3) awareness of the health benefits of fermented foods.

An online questionnaire was designed for the study, which consisted of closed and opened type of questions to assess the above mentioned areas of interest in addition to demographics. The questions were modeled based on those used in previously published studies on knowledge about and awareness of the health benefits of fermented foods [25–29]. Each question had multiple response options.

The first part of the questionnaire included respondent demographic data (gender, age, education, marital status, socioeconomic status, place of residence and overall health). The second part contained questions about the respondents’ knowledge of fermentation (including the microorganisms involved) and fermented foods. The third part of the questionnaire assessed the consumption of fermented foods and the respondents’ attitudes toward fermented foods. The last part of the questionnaire consisted of questions about awareness of the health benefits of fermented foods.

After obtaining informed consent from all participants, we conducted an anonymous questionnaire survey using a Web survey tool, 1.KA. The questionnaire was distributed to study participants through direct personal contact, email, Facebook, Twitter, and other social media applications. Participation in the study was completely voluntary, and participants could decline to participate in the study at any time. Our goal was to include at least 300 participants to achieve a statistical power of over 80% and a small statistical margin of error. For this reason, data collection took place in an electronic database from January to March 2021. We received 367 questionnaires, however, 18 of them were excluded due to missing data, hence the final sample size included 349 participants.

The study was approved by the Ethic Committee of Faculty of Health Sciences University of Maribor (038/2020/1786–04/504).

### Statistical analysis

Descriptive statistics were used to characterize participant demographics. Data were presented by frequencies and percentages for categorical variables, or by means and standard deviations for continuous variables. Variability around mean values was measured by standard deviations.

Only fully completed questionnaires were included in the analyses. Binary logistic regression was used as the main analytical tool to identify associations between the variables. The dependent variable in the model was home preparation of fermented foods. The seven independent variables were: participant characteristics (such as gender and age), formal education level, employment, socioeconomic status, place of residence, and presence of chronic health problems. Results are presented as odds ratios with 95% confidence intervals and *p*-values. Following Hsieh [30], a total number of 349 participants was calculated to have greater than 95% power for detecting a significant association for logistic regression (using alpha of 0.05, a total proportion of events of 0.3, and median odds ratio of approximately 2.5 to 1).

Statistical analyses were performed using IBM SPSS ver.27.0 (IBM Corp., Armonk, NY). Statistical significance was set at *p* < 0.05.

## Results

Of the 367 participants who gave their consent, 349 were included in the study (95.1%). As shown in Table 1, 84.0% of the participants were female and 16.0% of were male. The average age of participants included in the study was 31.6 ± 15.7 years; for men, it was 33.5 ± 15.7 years and for women, 30.9 ± 15.5 years, ranging from the youngest participant aged 16 years to the oldest aged 89 years. Most participants (*n* = 280; 80.2%) were from northeast Slovenia, whereas 69 participants (19.8%) were from other Slovenian regions.

**Table 1:** Characteristics of the study population

**Characteristics**	*N* = 349	%
Gender		
Male	56	16.0
Female	293	84.0
Age		
≤ 20	89	25.5
21—30	137	39.3
31—40	35	10.0
41—50	41	11.7
≥ 51	47	13.5
Education		
Primary school	60	17.2
High school	101	28.9
Vocational school	34	9.7
University	90	25.8
Master’s degree or higher	64	18.3
Marital status		
Married or civil union	122	35.2
Single	226	64.8
Monthly income (EUR)		
≤ 840	173	49.6
841 – 1.260	45	12.9
1.261 – 1.680	49	14.0
1.681 – 2.380	48	13.8
≥ 2.380	34	9.7
Place of residence		
Urban	131	37.5
Rural	218	62.5
Employment status		
High school pupil	64	18.3
Student	109	31.2
Employed	132	37.8
Unemployed	17	4.9
Retired	27	7.7
Chronic disease		
No	300	86.0
Yes	49	14.0
Scientific knowledge of fermentation		
Yes	59	16.9
No	290	83.1
Preparing fermented foods at home		
Yes	104	29.8
No	245	70.2

*Legend: n* frequency, *%* percentage

As shown in Table 1, 60 (17.2%) of the participants had completed elementary school, 101 (28.9%) had completed high school, and 34 (9.7%) had completed vocational school. Bachelor’s degree was held by 90 (25.8%) of the participants, and a master degree or higher was held by 64 (18.3%) of the participants. More than half of the participants were single (*n* = 226; 64.8%) while 122 (35.2%) of them were married or living in a civil union. Analysis of monthly income shows that about half of the participants (*n* = 173; 49.6%) had a monthly income of up to 840 euros.

About 37.5% lived in an urban environment. The majority of participants were either students (*n* = 109; 31.2%) or employed (*n* = 132; 37.8%). Most participants denied having a chronic disease (*n* = 300; 86.0%). Finally, slightly less than one- third (29.8%) of participants attempted to prepare fermented foods at home, while 70.2% never attempted to ferment at home.

First, we checked whether the participants knew the definition of fermentation with a question consisting of four different statements about fermentation. The question was single answer question with only one correct answer – the definition of fermentation. Overall, 59 (16.9%) of the participants knew the definition of fermentation while 290 (83.1%) did not (Table 1).

**Fig. 1:**
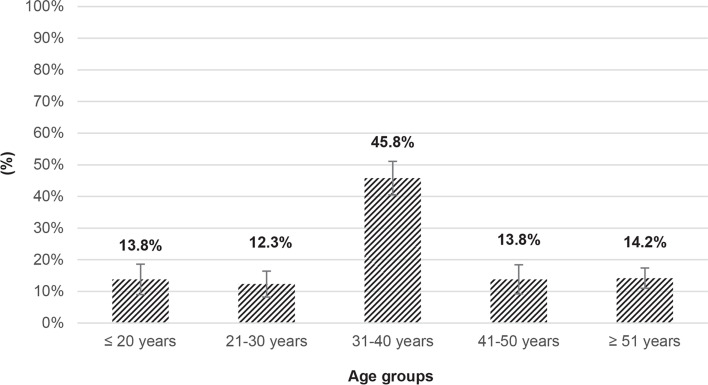
Knowledge of the fermentation process among the participants of the study. The X-axis represents the age group of the participants. The Y-axis represents the percentage of participants in each age group who answered the question correctly

The percentage of participants in each age group who answered the question correctly is shown in Fig. 1. The results showed that more adults (31—40 years) than younger (age groups ≤ 20 or 21 – 30 years) or older participants (age groups 41 – 50 or ≥ 50 years) were familiar with the definition of fermentation (*p* < 0.001) (Fig. 1).

We also investigated participants’ knowledge of the microorganisms involved in fermentation.

The results showed that 60.9% of participants included in the study had limited knowledge of the microorganisms involved in the fermentation process and were able to identify up to two groups of microorganisms (LAB and yeasts) while excluding parasites, viruses or pathogens from the process. About 18.3% of the participants showed better knowledge by being aware of the role of molds. About 11.8% of the participants had excellent knowledge of the microorganisms involved in fermentation and were able to identify all three groups of microorganisms involved (LAB, yeasts and molds) while knowing that parasites, viruses or pathogens were not involved in the process. Another 9% of the participants declared they did not know which microorganisms were involved in the fermentation process.

**Fig. 2:**
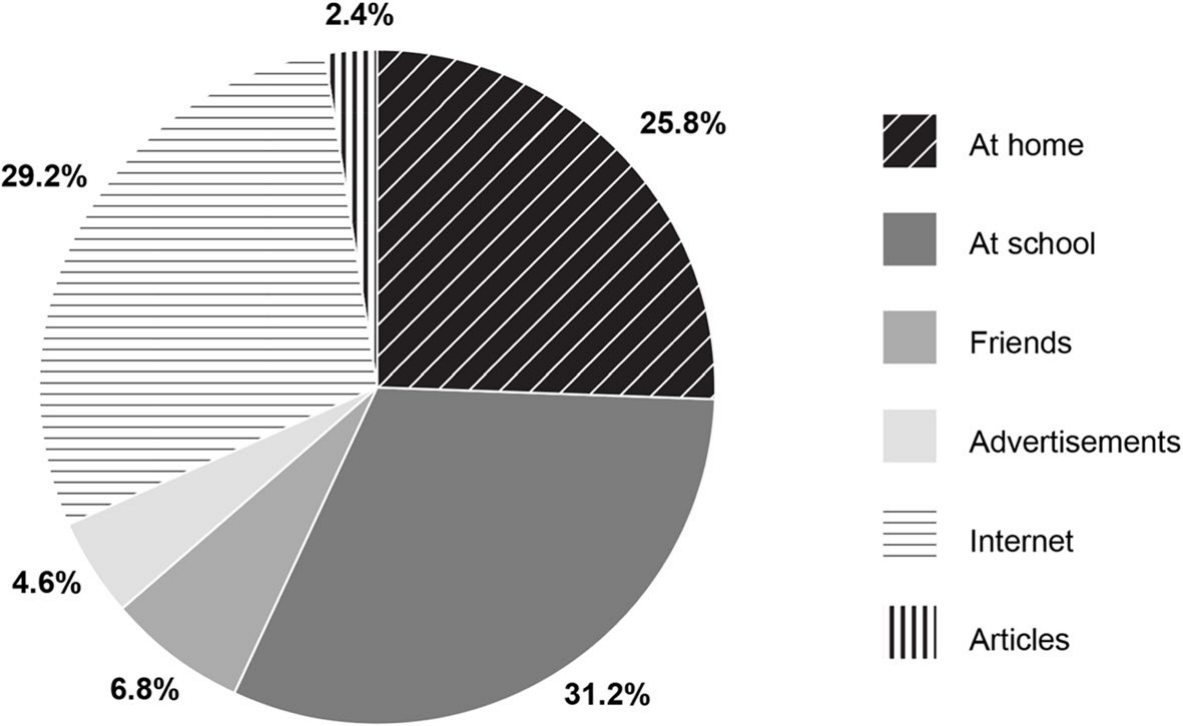
Acquaintance with fermented foods

As can be seen in Fig. 2, only 25.8% of the participants learned about fermented foods at home, 31.2% of the participants learned about fermented foods at school, while 29.2% of them learned about fermented foods on the Internet. A smaller proportion of participants learned about fermented foods through friends (6.8%), advertisements (4.6%), or articles (2.4%).

62.2% of participants were aware of the health benefits of fermented foods, especially in terms of their beneficial effects on gastrointestinal health (digestion and gut microbiota) and the immune system. In addition, 1.4% of participants disagreed with the health benefits of fermented foods, while 36.4% of participants stated that they were unaware of the health benefits of fermented foods.

Table 2 presents awareness of the specific health benefits of fermented foods. The results showed that participants were most aware of the health benefits of fermented foods for intestinal health, particularly for digestion (57.9%) and gut microbiota (49.0%), and for the immune system (43.6%). They were less aware of the health benefits of fermented foods for the skin (17.2%) or the cholesterol-lowering effects of fermented foods (19.1%). Participants were least aware of the possible beneficial effects of fermented foods on allergy (10.6%) and depression symptoms (10.6%). Interestingly, 296 (84.8%) of the participants would consume more fermented foods if they knew more about the health-related benefits of such foods.

**Table 2:** Study population awareness of health benefits of fermented foods

**Survey question**	*N* = 349	%
Do you know the health benefits of fermented foods?		
Yes	217	62.2
No	5	1.4
I don’t know	127	36.4
For the immune system		
Yes	152	43.6
No	197	56.4
For digestion		
Yes	202	57.9
No	147	42.1
For gut microbiota		
Yes	171	49.0
No	169	48.4
For skin		
Yes	60	17.2
No	256	73.4
For cholesterol		
Yes	63	19.1
No	266	80.9
For depression symptoms		
Yes	37	10.6
No	312	89.4
For diabetes		
Yes	41	11.7
No	308	88.3
For allergy		
Yes	37	10.6
No	312	89.4
Would you consume more fermented foods if you knew the health benefits?		
Yes	296	84.8
No	53	15.2

*Legend: n* frequency, *%* percentage

The frequency and types of fermented foods consumed by participants in our study are shown in Table 3. The results show that yoghurt was the most frequently consumed on daily basis compared to other fermented foods. Thirty-three (9.5%) of the participants consumed yoghurt daily and most of them consumed it several times a week (*n* = 84; 24.1%). Fresh and semi-hard cheeses were consumed by 159 (46.4%) and 183 (52.4%) participants several times per week, respectively. Hard cheese was consumed by almost half of the participants (*n* = 166; 47.6%) only once a month. Of all fermented foods, kombucha and water kefir were used the least, as 290 (83.9%) and 292 (83.7%) participants, respectively, reported never consuming them. Most participants used sour turnip (*n* = 83; 23.8%) and sauerkraut (*n* = 177; 50.7%) only seasonally. This was an interesting finding, as both sauerkraut and sour turnip are available in supermarkets in Slovenia throughout the year.

**Table 3:** Frequency and types of fermented food consumed by respondents

	Daily *n* (%)	Several times per week *n* (%)	Once a week *n* (%)	Several times per month *n* (%)	Once a month *n* (%)	Rarely *n* (%)	Seasonally *n* (%)	Never *n* (%)
Yoghurt	33 (9.5)	84 (24.1)	49 (14.1)	31 (8.9)	13 (3.7)	29 (8.3)	1 (0.3)	12 (3.4)
Milk Kefir	11 (3.2)	20 (5.7)	18 (5.2)	19 (5.4)	114 (32.6)	66 (18.9)	7 (2.0)	94 (26.9)
Fresh cheeses (cottage cheese, mozzarella, etc.)	11 (3.2)	159 (46.4)	61 (17.5)	45 (12.9)	22 (6.3)	33 (9.5)	3 (0.9)	12 (3.4)
Semi-hard cheeses (Gouda, Cheddar, etc.)	20 (5.7)	183 (52.4)	74 (21.2)	32 (9.2)	11 (3.2)	19 (5.4)	1 (0.3)	9 (2.6)
Hard cheeses (Cheddar, Parmesan, etc.)	24 (6.9)	47 (13.5)	35 (10.0)	38 (10.9)	166 (47.6)	15 (4.2)	4 (1.1)	35 (10.0)
Blue cheeses (Gorgonzola, Roquefort, etc.)	1 (0.3)	5 (1.4)	13 (3.7)	17 (4.9)	27 (7.7)	97 (27.8)	1 (0.3)	115 (33.0)
Sour turnip	2 (0.6)	8 (2.3)	26 (7.4)	29 (8.3)	55 (15.8)	97 (27.8)	83 (23.8)	49 (14.0)
Sauerkraut	2 (0.6)	7 (2.0)	16 (4.6)	40 (11.5)	39 (11.2)	42 (12.0)	177 (50.7)	26 (7.4)
Sourdough bread	8 (2.3)	11 (3.2)	13 (3.7)	14 (4.0)	7 (2.0)	84 (24.1)	2 (0.6)	210 (60.2)
Water kefir	2 (0.6)	4 (1.1)	5 (1.4)	3 (0.9)	5 (1.4)	32 (9.2)	5 (1.4)	293 (83.9)
Kombucha	2 (0.6)	1 (0.3)	4 (1.1)	3 (0.9)	3 (0.9)	42 (12.0)	2 (0.6)	292 (83.7)
Wine	9 (2.6)	23 (6.6)	39 (11.2)	44 (12.6)	29 (8.3)	165 (47.3)	5 (1.4)	33 (9.5)
Beer	10 (2.9)	21 (6.0)	44 (12.6)	41 (11.7)	22 (6.3)	163 (46.7)	5 (1.4)	43 (12.3)

*Legend: n* frequency, *%* percentage

**Fig. 3:**
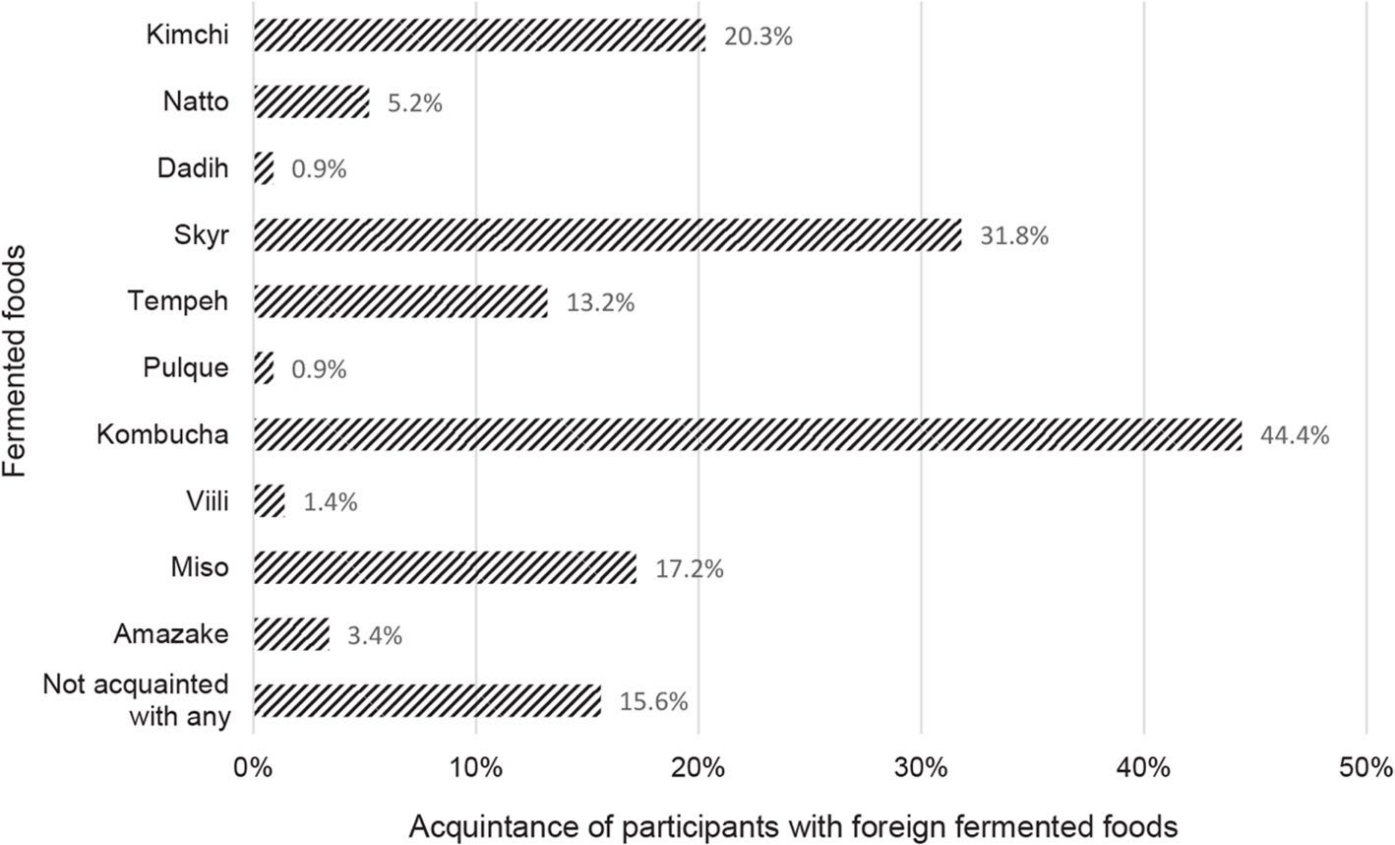
Acquaintance of study participants with the foreign fermented foods. Columns indicate the percentage of participants acquainted with a particular fermented food

Next, we assessed participants’ acquaintance with foreign fermented foods; the results are shown in Fig. 3. The results show that participants were most familiar with kombucha (*n* = 155; 44.4%), followed by skyr (*n* = 111; 31.8%) and kimchi (*n* = 71; 20.3%).

In addition, 119 (34.1%) participants tried at least one of the fermented foods mentioned (i.e., kimchi, natto, dadih, skyr, pulque, etc.) (Table 4).

**Table 4:** Consumption of foreign fermented foods

**Survey question**	*N* = 349	%
Have you tried any of the foreign fermented foods mentioned?		
Yes	119	34.1
No	230	65.9

*Legend: n* frequency, *%* percentage

Finally, Table 5 presents the associations between participant characteristics (i.e., age, gender, education, etc.) and home preparation of fermented foods. Our results show that the presence of chronic disease (OR = 2.81, 95%CI = 1.36–5.81, *p* = 0.004) is positively associated with the preparation of fermented foods at home. The explanatory variables included in the multivariable regression model explained 32.8% of the variation (Nagelkerke *R*^2^ = 0.328, model χ^2^ = 91.817, df = 19, *p* < 0.001).

**Table 5:** Multivariable associations between participant characteristics and preparing fermented foods at home

	OR (95% CI)	p
Age (years)		
≤ 20	1.00 (reference)	
21 do 30	1.76 (0.30–10.41	0.531
31—40	2.07 (0.28–15.18)	0.475
41—50	4.82 (0.67–34.84)	0.119
≥ 51	7.20 (0.89–58.45)	0.065
Gender		
Male	1.00 (reference)	
Female	0.51 (0.25–1.04)	0.062
Education		
Primary school	1.00 (reference)	
High school	0.62 (0.12–3.30)	0.573
Vocational school	1.14 (0.27–4.87)	0.857
University	2.08 (0.44–9.91)	0.356
Master’s degree or higher	4.18 (0.86–20.43)	0.077
Monthly income (EUR)		
≤ 840	1.00 (reference)	
841 – 1.260	2.10 (0.88–5.01)	0.094
1.261 – 1.680	1.65 (0.52–5.23)	0.397
1.681 – 2.380	0.84 (0.24–2.91)	0.781
≥ 2.380	1.64 (0.45–5.98)	0.451
Place of living		
Urban	1.00 (reference)	
Rural	1.37 (0.76–2.46)	0.293
Employment status		
High school pupil	1.00 (reference)	
Student	1.77 (0.27–11.63)	0.552
Employed	1.89 (0.21–16.77)	0.568
Unemployed	4.38 (0.48–39.79)	0.190
Retired	1,06 (0.09–12.43)	0.961
Chronic disease		
No	1.00 (reference)	
Yes	2.81 (1.36–5.81)	0.004

Nagelkerke *R*^2^ = 0.328,

*OR* Odds ratio, *95% CI* 95% confidence interval

When asked how they felt about fermented foods, 252 (72.2%) of the participants indicated that they felt safe when consuming fermented foods, and 230 (65.9%) of the participants had no fear before trying a new fermented food (Table 6). In addition, 259 (74.2%) of the participants thought that older people knew more about the fermentation process, and 258 (73.9%) of them expressed their interest in learning more about the process.

**Table 6:** Attitude of participants toward fermented foods

**Survey question**	*N* = 349	%
Do you feel safe when consuming fermented foods that contain live microorganisms?		
Yes	252	72.2
No	97	27.8
Are you afraid to try new fermented foods?		
Yes	119	34.1
No	230	65.9
Do you think older generations know more about fermentation?		
Yes	259	74.2
No	90	25.8
Would you like to know more about fermentation?		
Yes	258	73.9
No	91	26.1

*Legend: n* frequency, *%* percentage

## Discussion

Modern dietary patterns consisting of excess sugar, salt, and saturated fat, as well as unhealthy lifestyles, have contributed to global epidemics of type 2 diabetes mellitus, obesity, hormonal imbalances, depression, and mental health concerns around the world [10, 11]. Incorporating fermented foods into the diet could help with these lifestyle disorders, as there is now strong evidence of the effect of fermented foods on overall health [10]. The importance of the latter has motivated us to investigate and provide insight into the use and knowledge of the health benefits of fermented foods in Slovenia. As far as we know, this is the first study in Slovenia dealing with this topic, although we do not want to exclude the possibility that there are smaller, preliminary studies or projects whose results are not publicly available.

We included people of all ages, as children and young adults often assist in household fermentation practices and are involved in the process to some degree. We also included people regardless of their level of education, as it is often the case that people with lower levels of education and people living in rural areas know more about food preservation and practice it more often. In Slovenia, as in other countries, people have used fermentation to preserve and conserve food for thousands of years, even if they did not know much about the process and used fermentation to preserve food and increase its nutritional value according to a “recipe” they had in their family for generations. This has led to an extensive and heterogeneous list of fermented foods following traditions and cultural preferences in different geographic areas around the world [10, 31]. Children in Slovenia grew up eating local fermented foods, including sauerkraut, sour turnip and sour milk in rural areas, etc. They accepted them as part of their traditional dishes and learned to like them because they were told that they were good and healthy for them. Moreover, fermentation was of great importance in Slovenia during the famines in the first half of the last century. More recently, renewed interest in the health-promoting potential of fermented foods, especially in developed countries, has motivated many people to try preparing fermented foods at home (most commonly kombucha, kefir, sourdough, etc.) and to make fermented foods part of their diet [32].

Our results showed that although participants reported being familiar with the term fermentation and using it in their daily lives, less than one-fifth of participants knew the correct definition of fermentation. Our results are consistent with the findings of the study by Hekmat and Koba [28], in which almost two-thirds of 233 students at the College of Brescia were unfamiliar with the term fermentation. In the same study, about the same percentage was unsure whether cultured dairy products were fermented [28]. In our study, participants were mostly aware of the role of lactic acid bacteria, which is not surprising since they are commonly referred to as probiotics and are heavily promoted, especially for intestinal health [33]. Our study population demonstrated a better knowledge of the presence and role of LAB in fermented foods than the population in the study by Suahoo et al. [25]. In addition, our respondents were aware of the role of yeasts used in the fermentation of alcoholic beverages such as wine, beer, and cider. The latter are traditionally performed with Saccharomyces cerevisiae strains, the most common and commercially available yeast [34]. However, most respondents were unaware of the role of molds used in the production of many Asian fermented foods (e.g., Aspergillus oryzae and Aspergillus sojae) and in the aging of cheeses (e.g., Penicillium camemberti and Penicillium roqueforti) [35].

A generational difference was evident in the understanding of the fermentation process. Our respondents in their 30 s were the age group most likely to have the correct understanding and knowledge of the process. This age group and the younger participants were also the ones who were familiar with and tried various foreign fermented foods (e.g., kimchi, natto, skyr, etc.). This is not so surprising, as many commercially produced varieties of fermented foods that were once rather unknown in our region are now widely available, including kefir, kimchi, skyr, miso, tempeh, kombucha, etc. Our findings are in contrast to Sahoo et al. [25], where the middle or older age group prefer to consume fermented foods compared to the younger age group. The food market for fermented products has proven to be increasingly popular and growing its offer responding to the global trend. Our study participants, especially the younger ones, are predominantly not afraid to try new fermented foods, they are looking for new taste and texture experiences and experimenting with international cuisines. This is reflected in the finding that only about a quarter of our participants have become acquainted with preparing fermented foods at home. More participants became acquainted with fermented foods at school, on the Internet, or through advertisements. On the other hand, important aspects of fermented food consumption by retired people compared to younger respondents were observed by Camire et al. [26], with retired respondents having a greater interest in food fermentation due to a limited food budget and food insecurity, interest in health-promoting foods, or availability of more time for food preservation.

Regular consumption of yogurt is often included in dietary guidelines, but recommendations for other fermented foods are usually lacking [36]. In Slovenia, however, the most recent dietary guidelines and food pyramid recommend consumption of fermented foods such as sauerkraut and sour turnip in addition to yogurt and cheese, although the term “fermented foods” is not used [37].

Yogurt and other cultured dairy products are generally perceived by consumers as a good source of live and health-promoting organisms [38]. In our study, yogurt, fresh and semi-hard cheese were most commonly consumed by respondents (i.e., several times per week). Both yogurt and soft cheese serve as a source of live bacteria, as many studies have shown that these products contain relevant amounts of various LAB, ranging from 10^4^ to 10^9^ cfu/g [36]. Milk kefir and hard cheese were consumed less frequently by the respondents, while sauerkraut and sour tournip were consumed seasonally by most respondents. In addition, blue cheese, sourdough bread, water kefir, and kombucha were the least consumed of the fermented foods.

Recently, a new trend has been established towards minimally processed foods that can have a positive effect on our physical and mental well-being. In this context, fermented foods with potential health benefits gained much attention [6]. Because of these health-promoting properties, many people are motivated to try, consume, and even prepare fermented foods at home. Our results showed that almost one-third of the study participants were preparing fermented foods at home. We subsequently assessed the opinion of participants regarding the health benefits of fermented foods. More than half of the participants were aware of the health benefits of fermented foods, especially in terms of their positive effects on intestinal health and the immune system. Less was known about the potential positive effects of fermented foods on allergy and depression symptoms. This lack of knowledge about the potential benefits of fermented foods in other domains of human health is an area for improvement. This is especially important considering that more than 80% of study population declared they would consume more fermented foods if they knew more about the health benefits of such foods. Similarly, the study by Sobharani Devi et al. [29] found that although respondents preferred fermented foods in their diets, they lacked knowledge about fermented foods and their health benefits. However, participants in our study were much more knowledgeable about the health benefits of fermented foods than in the study by Sahoo et al. [25].

The ability of diet to modulate the gastrointestinal microbiota, improve or prevent dysbiosis, and improve human health is now well established [39–41]. In addition to supplementation with probiotics and food components known to influence the composition of the gut microbiota, fermentable fibers and prebiotics that enrich certain members of the gut microbiota [36]. Numerous studies have demonstrated the potential health benefits of fermented foods associated with either improved health or reduced disease risk [27, 42–45]. For this reason, consumption of fermented foods should be encouraged, as it may be the easiest way to introduce beneficial microorganisms into the gastrointestinal tract and represents an investment in future health.

## Conclusion and recommendation

It can be concluded that despite the fact that less than one fifth of the participants were familiar with the definition of fermentation and understood the process, almost one-third of the participants at least tried to ferment at home. Respondents who suffered from a chronic illness were more likely to ferment at home, presumably due to the supposed health benefits of fermented foods. Nowadays, families tend to follow a modern/Western diet and lifestyle, mostly due to lack of time and knowledge about the value of fermented foods and their preparation. This is reflected in the fact that only about a quater of the participants learned about fermented foods at home. Although more than half of the participants knew about the health benefits of fermented foods, these were mainly related to intestinal health and the immune system. The majority of respondents felt safe when consuming fermented foods and were not afraid to try new fermented foods. It is also noted that the knowledge about fermentation was not passed on to the younger generations as they had less knowledge about the process. Steps should be taken to educate younger generations about the health benefits of fermented foods, especially since most of them expressed interest in learning more about the process. Fermented foods should once again become part of the diet to improve overall health and reduce or postpone the onset of a variety of chronic diseases that plague us today.

## Data Availability

The datasets used and analysed during the current study are available from the corresponding author on reasonable request.
